# Damage associated molecular patterns and neutrophil extracellular traps in acute pancreatitis

**DOI:** 10.3389/fcimb.2022.927193

**Published:** 2022-08-12

**Authors:** Xiaoying Zhou, Shengchun Jin, Jingyi Pan, Qingyi Lin, Shaopeng Yang, Peter C. Ambe, Zarrin Basharat, Vincent Zimmer, Wei Wang, Wandong Hong

**Affiliations:** ^1^ Department of Gastroenterology and Hepatology, The First Affiliated Hospital of Wenzhou Medical University, Wenzhou, China; ^2^ School of the First Clinical Medical Sciences, Wenzhou Medical University, Wenzhou, China; ^3^ Department of General Surgery, Visceral Surgery and Coloproctology, Vinzenz-Pallotti-Hospital Bensberg, Bensberg, Germany; ^4^ Jamil-ur-Rahman Center for Genome Research, Dr. Panjwani Centre for Molecular Medicine and Drug Research, International Center for Chemical and Biological Sciences, University of Karachi, Karachi, Pakistan; ^5^ Department of Medicine, Marienhausklinik St. Josef Kohlhof, Neunkirchen, Germany; ^6^ Department of Medicine II, Saarland University Medical Center, Saarland University, Homburg, Germany; ^7^ School of Mental Health, Wenzhou Medical University, Wenzhou, China; ^8^ Zhejiang Provincial Clinical Research Center for Mental Disorders, The Affiliated Wenzhou Kangning Hospital, Wenzhou Medical University, Wenzhou, China

**Keywords:** DAMPs (damage-associated molecular patterns), NETs (neutrophil extracellular traps), acute pancreatitis (AP), HMGB1 (high mobility group box 1), HSP (heat shock protein), histone

## Abstract

Previous researches have emphasized a trypsin-centered theory of acute pancreatitis (AP) for more than a century. With additional studies into the pathogenesis of AP, new mechanisms have been explored. Among them, the role of immune response bears great importance. Pro-inflammatory substances, especially damage-associated molecular patterns (DAMPs), play an essential role in activating, signaling, and steering inflammation. Meanwhile, activated neutrophils attach great importance to the immune defense by forming neutrophil extracellular traps (NETs), which cause ductal obstruction, premature trypsinogen activation, and modulate inflammation. In this review, we discuss the latest advances in understanding the pathological role of DAMPs and NETs in AP and shed light on the flexible crosstalk between these vital inflammatory mediators. We, then highlight the potentially promising treatment for AP targeting DAMPs and NETs, with a focus on novel insights into the mechanism, diagnosis, and management of AP.

## Introduction

Acute pancreatitis (AP) is an acute inflammation of the exocrine pancreas and represents one of the most common gastrointestinal diseases, leading to acute admission to the hospital. The annual incidence in high-income countries reaches around 34 per 100,000 persons/year (Xiao et al., 2016). In the USA alone, the annual cost of treatment reaches $9.3 billion and the average hospitalization cost reaches $6,240 per patient (Mederos et al., 2021; Peery et al., 2021). AP is a complex disease that varies in severity and clinical course, from a self-limited course that resolves in a few days/weeks to severe conditions which result in systemic inflammatory response syndrome–associated extrapancreatic organ failure and even death (Habtezion et al., 2019). The past decades have witnessed more significant advances in understanding the pathology and mechanism of AP and some potentially promising therapeutic approaches to reduce morbidity and treatment costs (Peery et al., 2021). Nevertheless, still, no effective therapeutic agents currently exist to treat or prevent AP (Lee and Papachristou, 2019). Therefore, deeper and more evidential insights, as well as investigation into the mechanisms of AP, are urgently required.

Since Chiari observed autolysis of the pancreas in an autopsy study in 1896, a trypsin-centered theory of AP has been proposed and investigated for more than a century (Saluja et al., 2019). However, further in-depth exploration and the use of more effective research tools and models have enabled the discovery of other mechanisms underlying AP, including pathological calcium signaling and endoplasmic reticulum (ER) stress (Lee and Papachristou, 2019). Pathological calcium signaling is historically linked with early-stage pancreatitis and, over the years, it has been more widely studied and become universally acknowledged for its inducing both pro-cell death and pro-inflammatory pathways in AP. This is typical for premature trypsinogen activation, activation of nuclear factor-κb (NF- κB), mitochondrial dysfunction and necrosis (Lee and Papachristou, 2019). Inositol trisphosphate receptor (IP3R) and ryanodine receptor (RyR) Ca^2+^ channel induction by the toxin, thus enhances the release of Ca^2+^ from the ER, causing the continued elevation of Ca^2+^ in the cytosol ([Ca^2+^]i) and mitochondria ([Ca^2+^]m) (Saluja et al., 2019). On the other hand, the rising concentration of [Ca^2+^]m induces the mitochondrial permeability transition pore (MPTP) and leads to the dysfunction of mitochondria (in producing ATP and reducing the cytosolic calcium), altogether resulting in the elevated concentration of calcium (Habtezion et al., 2019).

When it comes to the role of the immune system, “damage associated molecular patterns” (DAMPs), which are produced or released by injured, dying, or dead cells to activate signaling and sterile inflammation, have a significant role (Saluja et al., 2019). In 1994, Polly Matzinger proposed the “danger theory”, which claimed that distressed or damaged cells could release endogenous danger signals (Matzinger, 1994). Later, these danger signal substances were named “damage-associated molecular patterns” (DAMPs) by Land in 2003 (Land, 2003). In the next two decades, the significant role of DAMPs in the pathology of AP, which links the local tissue damage to systemic inflammation reaction syndrome (SIRS), became increasingly clear (Kang et al., 2014a). Aseptic inflammation is the initial manifestation of injury in AP, and numerous studies have shown that DAMP-mediated aseptic inflammation is the essential event mediating further pancreatic injury, downstream organ injury, and disease resolution, suggesting that DAMPs are an important factor in the initiation and perpetuation of AP (Hoque et al., 2012). Considering the vital roles of linking local tissue damage to systemic inflammation, DAMPs could be moieties of concern in the pathogenesis of AP, which function by recruiting inflammatory cells and activating adaptive immune responses (Kang et al., 2014b).

Neutrophils are primarily thought to be the first immune cells to be recruited to inflammatory tissues in the event of acute inflammation (Maas et al., 2018). *In vivo* evidence suggests that neutrophils are the first line of defense against bacterial and fungal infections (Wan et al., 2020). If activated, they perform various antimicrobial functions and tasks, including phagocytosis, cytokine secretion, and degranulation (Land, 2003). These cells kill invading pathogens with a large number of antibacterial agents, including reactive oxygen species (ROS) and hydrolases (Wan et al., 2020). Recently, the formation of so-called neutrophil extracellular traps (NETs) has been described as a new defense mechanism. NETs were first described in 1996 as a pathway of cellular death designated NETosis, different from apoptosis and necrosis (Takei et al., 1996), further detailed by Brinkmann et al. They demonstrated that after stimulation of isolated neutrophils with interleukin-8 (IL-8), a major neutrophil chemoattractant, bacterial lipopolysaccharide (LPS, a component of gram-negative bacteria) or phorbol 12-myristate 13-acetate (PMA, a potent activator of protein kinase C, PKC), NETs could be generated *in vitro* to create a physical barrier that prevents the spread of pathogens and facilitates killing microbes *via* high concentrations of antimicrobial proteins and phagocytosis (Brinkmann et al., 2004). Further studies, on the contrary, demonstrated the essential role of NETs in tissue damage, vaso-occlusion promotion, and sterile inflammation promotion (Papayannopoulos, 2018). Research has revealed the involvement of NETs in the pathogenesis of sepsis, connective tissue diseases, cardiovascular diseases, autoimmune diseases, and cancer (Döring et al., 2017; Lee et al., 2017; Bonaventura et al., 2020; Masucci et al., 2020; Klopf et al., 2021; Cristinziano et al., 2022). Its double-edged function is uncovered for its protective role in mediating host defense by trapping and killing microorganisms as well as the detrimental effect, when excessive NETs lead to tissue injury by facilitating thrombus formation, causing a “no-reflow” phenomenon and exacerbating hepatic ischemia reperfusion injury (Cahilog et al., 2020). Given these novel findings, a comprehensive investigation into the complex interaction between NETs and APs might be of special interest.

In this review, we introduce the pathology of DAMPs and NETs, and their interplay in the pathological progress of AP. Given the close relationship between DAMPs and NETs, the new approach targeting DAMPs and NETs could be promising and worth further investigation. Here we also discuss the existing potential therapeutic interventions targeting both DAMPs and NETs in AP and shed a light on the future strategy in the hope of more effective interventions at the early stage of AP.

## DAMPs are endogenous danger signals in AP

DAMPs are endogenous substances that are usually sequestered inside cells by a cell membrane or organelle membrane to play an intracellular physiological role through precise regulation. They are localized within the nucleus and cytoplasm (HMGB1), cytoplasm alone (S100 proteins), exosomes (exRNAs), oncosomes (HSPs), secretory lysosomes (ATP) and in plasma components, such as complement cascade elements C3a, C4a, and C5a (Tang et al., 2012). Once released (either passively or actively) by dead or dying cells, they can serve as endogenous danger signals to alert the innate immune system to unscheduled cell death, stimulate anti-microbial defense, and respond to stress (Lotze et al., 2007; Tang et al., 2012). Despite their essential role in tissue healing after inflammation, excessive exposure to DAMPs could lead to uncontrolled sterile inflammation and various diseases, like sepsis, diabetes mellitus, and chronic neurodegenerative disorders (Shin et al., 2015; Thundyil and Lim, 2015; Venereau et al., 2015; Denning et al., 2019a).

Former studies have shed light on the close association between DAMPs and AP, emphasizing their role as endogenous danger signals in AP (Liu et al., 2017; Wu et al., 2018b; Biberci Keskin et al., 2019). DAMPs initiate immune responses through the receptors which are divided into two types: pattern recognition receptors (PRR), which include Toll-like receptors, c-type lectin receptors, nod like receptor (NLR)-family pyrin domain-containing 3, Retinoic acid-inducible gene I (RIG-I)-like receptors, cytoplasmic DNA sensors as well as non-PRR DAMP receptors, which include receptors for advanced glycation end (RAGE) products and trigger receptors expressed on myeloid cells, G-protein-coupled receptors and ion channels (Gong et al., 2020). The detailed functions of those receptors and the corresponding DAMPs in AP are outlined in **Table 1** and further discussed below.

**Table 1:** DAMPs receptors, associated DAMPs, expression pattern and their effects related to AP.

DAMP receptors	DAMPs	Expression pattern	Main effect	Refs
TLR		Ubiquitous, high in immune cells	Promote the expression of pro-inflammatory genes, thus upregulate the production of cytokines, chemokines, and adhesion molecules.	(Yu et al., 2010; Lin et al., 2011; Vidya et al., 2018; Gong et al., 2020)
TLR2	HMGB1, HSP60, HSP70, histone			
TLR4	HMGB1, HSP22, HSP60, HSP70, HSP72, histone			
TLR9	DNA, HMGB1			
NLRP3	ATP	DCs, neutrophils, monocytes and macrophages	Promote the activation of caspase-1. Increase the secretion of IL-1β and IL-18. Initiate pyroptosis.	(Sutterwala et al., 2006; Jin and Flavell, 2010; Mangan et al., 2018; Gong et al., 2020)
RAGE	HMGB1	Ubiquitous, high in T cells, B cells, and macrophages	Promote the expression of pro-inflammatory genes. Mediate cell migrationand apoptosis.	(Chuah et al., 2013; Hudson and Lippman, 2018; Gong et al., 2020)
P2X7R (G protein-coupled receptor)	ATP	Ubiquitous	Promote the release of cytokine and chemokine, the activation of NLRP3 inflammasome, transcription factor and T cells.	(Di Virgilio et al., 2017b; Adinolfi et al., 2018; Martínez-García et al., 2019; Gong et al., 2020)
P2Y2R (ion channel)	ATP	Ubiquitous, high in immune cells, epithelial and endothelial cells	Promote the migration, and activation of immune cells. Control iron channels.	(Xu et al., 2018; Gong et al., 2020)

## DAMPs and AP

DAMPs released or exposed from dying or dead cells play an important role in the pathogenesis of AP by linking local tissue damage to systemic inflammation. With inflammatory response as the central link, we divided the pathological mechanism of DAMPs in AP into the following five aspects, which will be expounded later.

1. Inducing production and release of inflammatory cytokines, interferons, chemokines and cell adhesion molecules (West and Shadel, 2017; Gong et al., 2020).

2. Mediating the formation of NLR Family Pyrin Domain Containing 3 (NLRP3) inflammasome (Liu et al., 2017).

3. Encompassing the activation and recruitment of innate immune cells, such as macrophages, dendritic cells and neutrophils (Kang et al., 2014a; Zhao et al., 2018; Habtezion et al., 2019)

4. Participating in the formation of NETs by activated neutrophils (Merza et al., 2015; Hu et al., 2020; Linders et al., 2020a).

5. Affecting the inflammatory process by participating in autophagy, necrosis, apoptosis and other pathways (Zhang et al., 2013; Yu et al., 2016; Gukovskaya et al., 2017).

### HMGB1 and AP

High mobility group box-1 (HMGB1) is one of the most prototypical DAMPs that has been well-studied for almost half a century. HMGB1, DNA chaperone, is an abundant and highly conserved nuclear protein that acts as a key determinant in reverse chromosomal DNA binding and bending to facilitate nucleosome formation and regulate gene events. A meta-analysis suggested that HMGB1 is an useful indicator of the degree of pancreatic inflammatory response from the healthy control, MAP patients to SAP patients (Li et al., 2018). In experimental AP models, HMGB1 levels decrease when inhibitors (such as pyrrolidine, dithiocarbamate) and neutralizing antibodies are used (Yasuda et al., 2007; Yang et al., 2008; Zhang et al., 2010b). Targeting the 3’ untranslated region of HMGB1, micro-RNA (miR)-340-5p downregulates HMGB1 expression and restrains the activation of Toll-like receptor 4 and enhanced protein kinase B (AKT) signaling, leading to subsequent inhibition of inflammation and apoptosis (Gao et al., 2022a). Taken together, these studies have established extracellular HMGB1 as a critical mediator in AP.

HMGB1 can be passively released by somatic cells in the process of the necrosis, when disrupting membrane integrity or actively secreted from regulated cell death processes, such as necroptosis, pyroptosis, ferroptosis, or apoptosis (Scaffidi et al., 2002; Jiang et al., 2007; Kaczmarek et al., 2013; Lu et al., 2014; Hou et al., 2018; Murao et al., 2021a). Similarly, it can be actively excreted by either immune cells (such as monocytes) or non-immune cells (such as epithelial cells) (Shen and Li, 2015). HMGB1 signals through RAGE and *via* distinct toll-like receptors (TLR), e.g. TLR2 and TLR4 (Gong et al., 2020). MiR-181a-5p/HMGB1/TLR4EV, a new signaling pathway, was proposed by (Liu et al., 2021), suggesting that the encapsulated MALAT1 (Metastasis associated lung adenocarcinoma transcript 1) competitively binds to miR-181a-5p, thus inhibiting HMGB1 induced TLR4 signaling pathway, inducing M1 polarization of macrophages in AP, thereby promoting the release of IL-6 and tumor necrosis factor (TNF)-α (Liu et al., 2021).

**Figure 1:**
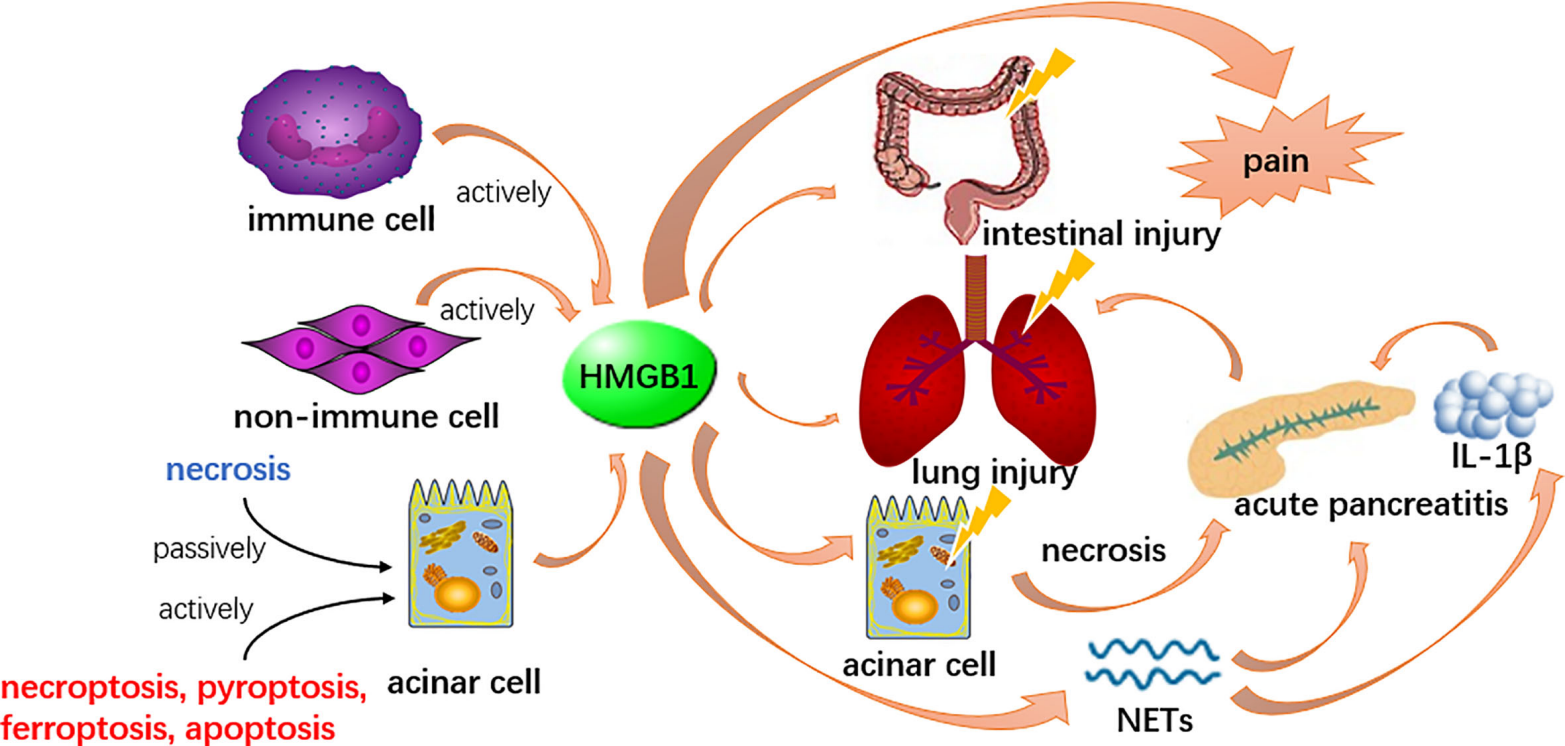
HMGB1 could be released by acinar cells and other cells in both active and passive ways. It participates in the formation of NETs and induce other acinar cell necrosis to boost the inflammation, therefore enhance the pathological process of AP. Meanwhile, HMGB1 is involved in lung and intestinal injury secondary to AP and serves as a pain mediator.

The action of HMGB1 appears to differ from localization. The release of extracellular pancreatic HMGB1 could be a central event in early stage of pancreatitis **(** **Figure 1** **)**, potentially by inducing autophagy resulting in necrosis when widely participate in numerous biological processes, including the formation of the chromosomal protein glycyl lysine isopeptide cross-link, and the positive regulation of phosphorylation, protein acid phosphorylation, the phosphate metabolic process and the phosphorus metabolic process (Gao et al., 2017; Gukovskaya et al., 2017). Likewise, HMGB1 might also serve as a late inflammatory factor to stimulate NF-κB nuclear translocation thereby enhancing inflammatory cells (such as the monocytes, neutrophils and dendritic cells) positive regulating the release of inflammatory cytokines such as IL-1α, IL-1β, TNF-α, etc., thus, act as an instrumental mediator in amplifying and maintaining the inflammatory cascade (Yu et al., 2015). Recent evidence indicates that HMGB1 participates in pancreatic, intestinal and lung injury during AP (Luan et al., 2013; Chen et al., 2017; Huang et al., 2019). Based on these effects, some new therapeutic approaches, such as calycosin and mesenteric lymph duct ligation to alleviate acute lung injury and euphorbia kansui to restore intestinal mucosa in severe AP were recently studied (Huang et al., 2021; Qiu et al., 2021; Tang et al., 2021; Zhu et al., 2021). In an experimental AP model, macrophage-derived HMGB1 served as a pain mediator in the early stage of AP (Irie et al., 2017). A recent study claims HMGB1 might induce AP through activation of NET and subsequent production of IL-1β, which may offer therapeutic targets for inflammation suppression (Irie et al., 2017). On the contrary, endogenous pancreatic HMGB1 may have an anti-inflammatory effect for its action in enhancing cell survival by increasing autophagic flux (Tang et al., 2010). This thought was supported by a study in which the observation of endogenous pancreatic HMGB1 deficient mice by the knockout gene of HMGB1 resulted in accelerated tissue injury and high mortality in AP (Kang et al., 2014b).

### Heat shock proteins (HSPs) and AP

Heat shock proteins (HSPs) are conserved proteins, mainly involved in protein folding and maturation (Wu et al., 2017). The major groups of HSPs are classified based on the weights of molecules, including HSP27, HSP40, HSP60, HSP70, HSP72, HSP90, HSP100 and HSP110 (Zininga et al., 2018). As molecular chaperones, HSPs play an important cytoprotective role and their expressions are induced under stressful conditions such as heat shock (Wu et al., 2017; Narayanankutty et al., 2019). HSPs were initially found intracellular, although extracellular HSPs, also representing DAMPs, were discovered later, which have been found in extracellular vehicles such as exosomes and oncosomes, on membrane surfaces or acting as free HSPs under various pathological conditions (Taha et al., 2019).

It has been reported that intracellular HSPs are upregulated in AP models, including the arginine, dibutyltin dichloride, and cerulein-induced AP models, and the high level of HSPs may have a protective effect against tissue damage (Feng and Li, 2010). In the same direction, heat shock factor protein 1 (HSF-1) knockout mice with defects in HSPs synthesis were reported to be more severely affected by cerulein-induced pancreatitis (Bhagat et al., 2002).

Intracellular HSP27, HSP60, and HSP70 are direct effectors in mediating a protective effect against AP (Kang et al., 2014a), by inhibiting trypsinogen activation (Lee et al., 2000; Giri et al., 2017), modulating NF-κB signaling (Giri et al., 2017), preventing proinflammatory cytokines (Meng et al., 2013), reducing autophagy (Kim et al., 2011), preserving the actin cytoskeleton (Kubisch et al., 2004), aiding in cytoskeletal recovery and limiting oxidative damage (Ethridge et al., 2000). Many studies have also found that enhancing the function of those three types of intracellular HSPs can protect against AP in mice by using co-inducers, that promote the expression or introduction of HSPs (Rakonczay et al., 2002; Szabolcs et al., 2009; Li et al., 2021a). This could be a future research subject and a potential treatment target for AP in humans.

Although the transgenic overexpression or specific pre-induction of Hsp72 failed to protect against cerulein-induced pancreatitis, it did accelerate tissue recovery, possibly through an attenuated NF-κB signaling (Rakonczay et al., 2003; Lunova et al., 2012). This might also represent a potential future treatment strategy.

While intracellular HSPs function as chaperones to assist with biosynthetic pathways, extracellular HSPs released from damaged cells that are generally dying, following apoptosis, necrosis, and cellular stress can function as alarm signals to induce inflammation through activation of TLR2, TLR4, and cluster of differentiation (CD)91 (Schaefer, 2014; Roh and Sohn, 2018; Taha et al., 2019).

Song et al. found out that extracellular HSP70 may induce SIRS-like reactions to aggravate cerulein-induced pancreatitis through TLR-4 in mice by the administration of recombinant HSP70 (Song et al., 2008). In addition, low serum HSP70 levels are associated with poor prognosis in AP patients (Arriaga-Pizano et al., 2018). Budvytyte et al. discussed these two findings above, as they found out the serum HSP90 levels increased linearly with increasing AP severity while the relationship was not linear in the case of HSP70 (Budvytyte et al., 2021). The mechanisms of extracellular HSP in AP are not yet conclusively elucidated, so further investigations are still required.

### DNA and AP

The study from Gornik et al. showed that serum free DNA was relevant to AP and its severity (Gornik et al., 2009). DNA methylation patterns based on plasma DNA have been developed as a new model to predict the severity of AP (Sun et al., 2021). During cell damage, nuclear and mitochondrial DNA leaks into the blood and activates the immune system, leading to a variety of diseases, including multiple organ dysfunction, failure to respond to sepsis or trauma, neurodegenerative diseases, etc. (Zhang et al., 2013).

The main source of free serum DNA in AP may be cell death (apoptosis or necrosis) or necrosis of pancreatic tissue (Gornik et al., 2009). The mechanism of DNA release into the circulation has not been elucidated, but the release of DNA from neutrophils *via* NETosis may be one way in which it is released into the circulation (Gould et al., 2015). Neutrophils release DNA by forming NETs which could aggravate inflammation of AP and promote pancreatic duct obstruction (Murthy et al., 2019; Goggs et al., 2020; Hu et al., 2020; Shi et al., 2020). The DNA released by dead and necrotic cells is also a typical DAMP (Habtezion et al., 2019), and it has been proved that DNA released from necrotic cells could be an effective activator of the innate immune system, including dendritic cells and macrophages (Zhao et al., 2018; Habtezion et al., 2019).

**Figure 2:**
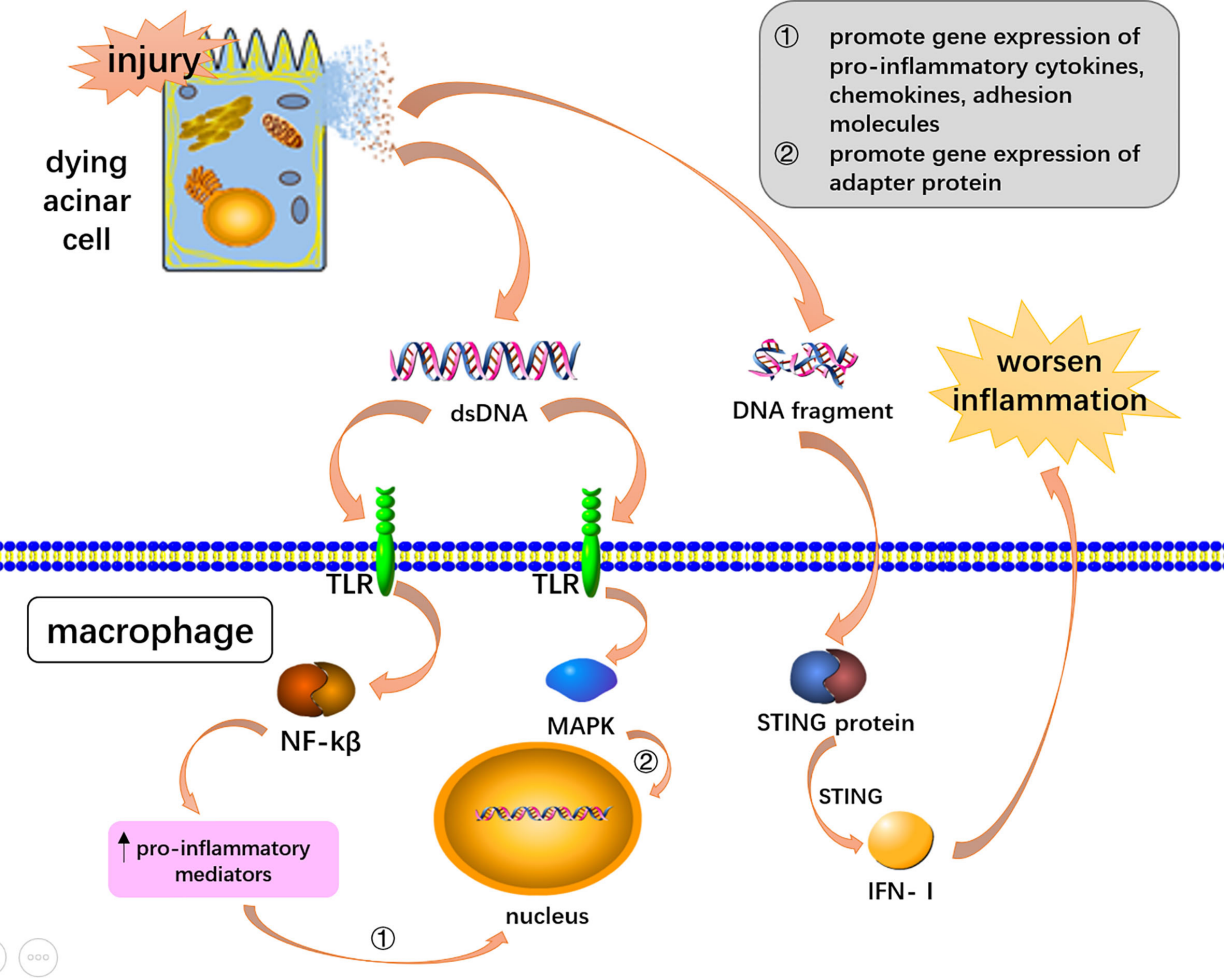
DNA mediated inflammation *via* DAMPs receptors and STING signaling. Double-stranded DNA and DNA fragments were released from dying acinar cells. DsDNA activated the NF-KB and MAPK pathway through toll-like receptors to induce AP-associated inflammation. DNA fragments worsen AP-associated inflammation through STING signaling pathway in macrophages.

STING (stimulator of the interferon gene) and apoptotic and necrotic DNA fragments are recognized by STING’s unique receptor function (Song et al., 2008). When acute pancreatitis occurs, the expression of STING protein can activate downstream signaling pathways and promote inflammation (Sundar et al., 2021). Studies have found that STING is associated with AP, in which the DNA released by the dying acinar cells activates STING in macrophages, and STING drives the formation of pro-inflammatory cytokines and type I interferon to worsen AP-associated inflammation (Palmai-Pallag and Bachrati, 2014; West and Shadel, 2017; Picca et al., 2018; Zhao et al., 2018; Sundar et al., 2021) **(** **Figure 2** **)**. Preventing the accumulation of their own DNA and blocking the over-activation of STING signals to reduce the generation of pro-inflammatory factors provides an attractive direction for future drug development (Ahn and Barber, 2014).

Toll-like receptors, such as TLR9 could recognize DNA to exacerbate inflammation in AP (Palmai-Pallag and Bachrati, 2014; Patel, 2018; Habtezion et al., 2019). The mechanism of double-stranded DNA could be toll-like receptor activation of the NF-kB signaling pathway (Lee and Papachristou, 2019). TLR9 recognition signals activate mitogen-activated protein kinases (MAPKs) and NF-κB to induce inflammatory downstreaming passways (Palmai-Pallag and Bachrati, 2014; West and Shadel, 2017; Barrera et al., 2021). The signaling pathway promotes gene expression of pro-inflammatory cytokines, chemokines, and adhesion molecules, as well as triggers inflammation through adaptor myeloid cell differentiation for major reactive protein 88 (MYD88) (Palmai-Pallag and Bachrati, 2014; West and Shadel, 2017; Lee and Papachristou, 2019; Barrera et al., 2021) **(** **Figure 2** **)**. Animal experiments carried out by Hoque et al. demonstrated that TLR-9 receptor antagonists can significantly reduce pancreatic edema and inflammatory cell infiltration, highlighting new therapeutic avenues for AP (Hoque et al., 2011).

Mitochondrial DNA (mtDNA) is one of the mitochondrial DAMPs (West and Shadel, 2017; Patel, 2018; Barrera et al., 2021), which is released when mitochondria are dysfunctional or damaged (Barrera et al., 2021). Research by Kocsis et al. supports signaling *via* plasma DNA, and increased plasma DNA concentration is correlated to AP and its severity (Kocsis et al., 2009). The elevation of plasma mtDNA in AP patients could serve as a precise early predictive index of pancreatic necrosis (Wu et al., 2018b). MtDNA can cause inflammation through hypomethylated CpG motifs similar to bacterial DNA and bacteria-like non-methylated CPG-rich motifs (Palmai-Pallag and Bachrati, 2014; Picca et al., 2018). MtDNA is released systemically by T/HS system and activates p38 MAPK in neutrophils, probably *via* TLR-9, which results in the development of an inflammatory phenotype (Zhang et al., 2010a) MtDNA can be activated by TLR9, NLRs, cyclic GMP–AMP (cGAS), and other innate sensors that lead to inflammatory responses (Palmai-Pallag and Bachrati, 2014; West and Shadel, 2017; Picca et al., 2018). MtDNA is an effective DAMPs due to its specific characteristics, take its unique structure, relative hypomethylation and its enhancive sensitivity to oxidative damage for example (West and Shadel, 2017).

### Histones and AP

Histones are the basic structural constituents of cellular chromatin. Eukaryotic DNA is surrounded by an octahedron of H2A, H2B, H3 and H4. The linker histone H1 facilitates further organization of chromatin. Except for their structural effects on chromosomes, the role and dynamics of chromosomes are also influenced by histone variants and the process of post-translational modifications (Campos and Reinberg, 2009; Biterge and Schneider, 2014). In addition to the physiological functions described above, when histones spill out of the nucleus, it can also serve as endogenous danger signs and DAMPs, playing an important role in the production and development of inflammation (Lu et al., 2014).

Histones can be released passively, for example, necrosis, necroptosis, apoptosis, and NETosis (Murao et al., 2021a). In particular, citrullinated histones are an important component of NETs and may be the mechanism by which the innate immune system exacerbates pancreatic injury (Thiam et al., 2020). In addition to passive released by cell death, histones can also be actively secreted through the exosomal exocytosis of living cells (Murao et al., 2021a).

**Figure 3:**
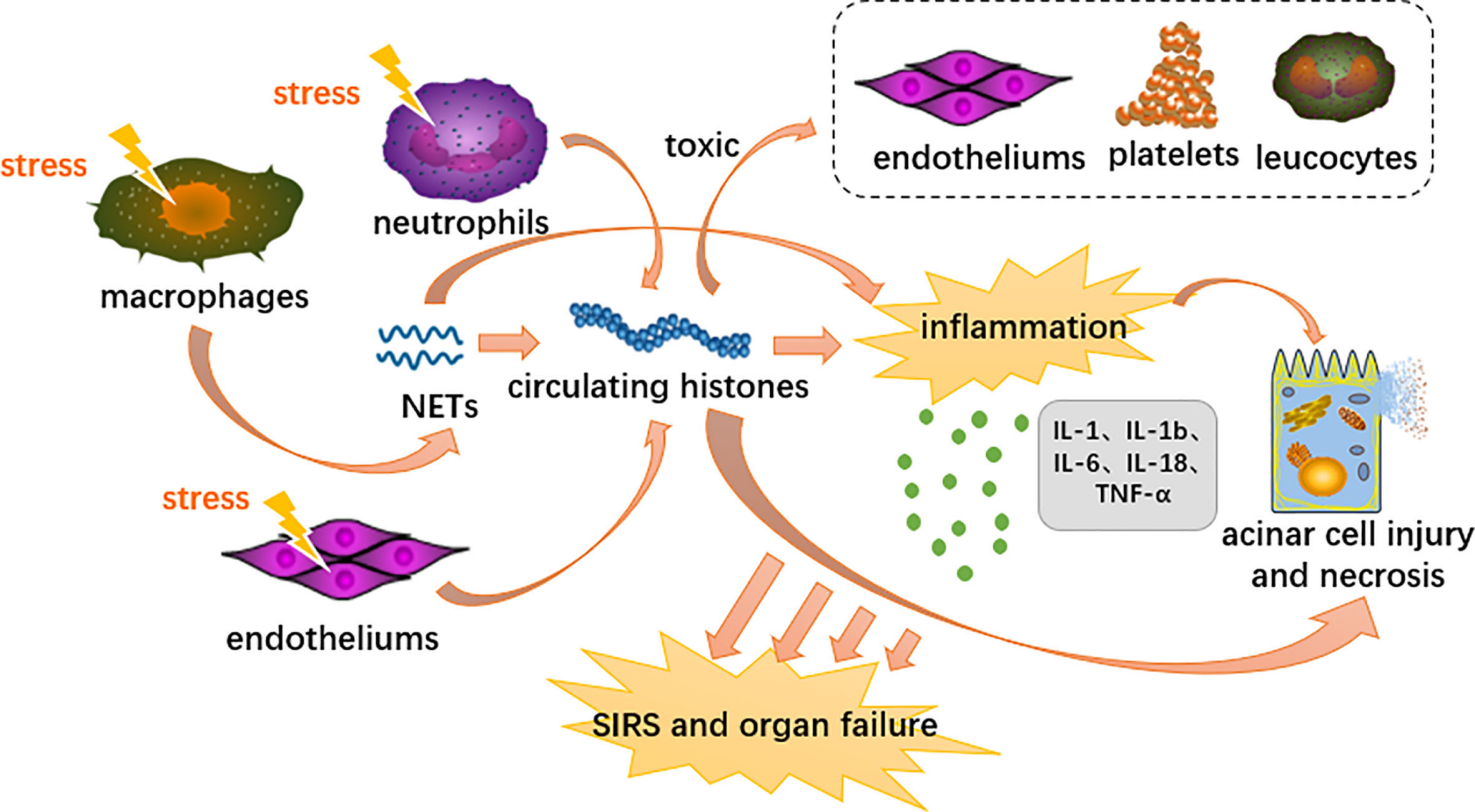
Proposed immunopathological roles of circulating histones in acute pancreatitis. DAMPs activate innate immune cells (neutrophils and macrophages) and endothelial cells through PRRs, triggering highly inflammatory programmed cell death such as neutrophil extracellular traps, necroptosis and necrosis. Extracellular histones mediate inflammation response, organ injury and death through TLR and NLRP3 inflammasome pathways.

Extracellular histones mediate sterile inflammatory responses, distant multiorgan damage and even death through activation of TLRs and the NLRP3 inflammasome signaling pathway (Semeraro et al., 2011; Allam et al., 2012; Li et al., 2021b). In terms of TLRs, histones specifically bind to and activate TLR2 and TLR4, which trigger MyD88 signaling followed by NF-κB and MAPK expression (Semeraro et al., 2011; Allam et al., 2014). Histones activate NLRP3 primarily through induction of intracellular oxidative stress (Allam et al., 2014). The NLRP3 inflammasome is likely a downstream component that integrates various indirect stimuli of IL-1β and IL-18 proteolysis (Allam et al., 2013) (**Figure 3**). Apart from the above two pathways, histones also have the pathogenic effects of bactericidal ability, cell toxicity, platelet activation, and protecting DNA degradation (Allam et al., 2014). In experiments where individual components of histones induced inflammatory cytokines in BMDC (bone marrow-derived dendritic cells), it was concluded that all histones had the ability to induce the production of TNF and IL-6 (Allam et al., 2012).

Researches showed that histones in the blood serve as DAMP and cause sterile inflammation leading to SIRS and organ failure in the process of AP (Kang et al., 2014a). At the cellular level, platelets, endothelial cells and leukocytes are also exposed to the toxic effects of extracellular histones. (Allam et al., 2014). Liu et al. further indicate that elevated levels of circulating histones are strongly associated with AP’s disease severity and mortality (Liu et al., 2017). Circulating histones accumulate in the inflamed pancreas and actively contribute to pancreatic acinar cell necrosis by destroying the plasma membrane in a burden- and dose-dependent manner (Liu et al., 2017; Szatmary et al., 2017; Biberci Keskin et al., 2019). In a previous experimental model of AP, we found a good correlation between elevated extracellular histones and pancreatic necrosis events. This is in addition to the concomitant damage to distant multiple organs, such as the heart, liver, lungs and kidneys, which are the most commonly affected organs (Guo et al., 2014; Ou et al., 2015). Circulating concentrations of nucleolytic degradation products reflect the extent of tissue damage and cell death. As the most abundant protein in the nucleus, histones can be used as a better biomarker to stratify the severity of a disease. (Ou et al., 2015).

Now that we know histone represents key mediators of AP, we might explore histone as a therapeutic target for treatment in the future. Negatively charged surface molecules (e.g., phosphatidylserine) enhance histone membrane interactions, so neutralizing the charge on circulating histones could be a promising therapeutic strategy (Szatmary et al., 2017). In mouse models, we found that anti-histone antibodies could be used to rescue mice from multi-organ failure due to histone injections (Xu et al., 2009; Xu et al., 2011; Li et al., 2021b). In subsequent experiments, the histone neutralizing antibody BWA3 halted histone related lesions, but it remains unclear exactly how it can block the physiological effects of circulating histones. (Monestier et al., 1993). Therefore, a potential therapeutic approach could be the development of anti-histone therapies to delay the second attack early in the process of AP (Kang et al., 2014a; Hartman et al., 2015; Liu et al., 2017). In addition to this, activated protein C serves as a serum protease, it can destroys histones that spill over into the extracellular space, thereby blocking the pathological processes associated with histones (Ammollo et al., 2011).

### ATP and AP

The pancreatic acinar cells are typical excitable exocrine cells featured by its high secretory turnover which is mainly supplied and closely dependent by mitochondrial production of ATP (Petersen and Tepikin, 2008). Given that ATP mainly kept in reserve at acinar cells with high concentration, the elevating extracellular concentration of ATP is thought to attribute to the release of injured cells (Yegutkin et al., 2006). Necrotic Acinar cells undergoing cytoplasmic membrane destruction are likely to be the main source of extracellular ATP (eATP) (Dixit et al., 2019b). Other sources of high eATP levels may include activated immune cells, duct cells, endothelial cells, or even cells undergoing apoptosis (Bodin and Burnstock, 1996; Elliott et al., 2009; Junger, 2011; Kowal et al., 2015).

The decrease of mitochondrial ATP production is the typical event at the early stage of AP. In normal physiological condition, Ca^2+^ is released from the ER and maintain a stable concentration to promote steady function as part of a signaling mechanism that stimulates the production of ATP in mitochondria (Criddle et al., 2007). The cellular Ca^2+^ concentration overload or other toxins (like bile acid, alcohol) cause mitochondrial permeability transition pores continue to open in a high-conductance state, resulting in the disorder of the membrane potential needed to generate ATP (Mukherjee et al., 2016). ATP depletion deteriorates and perpetuates the toxic overload of Ca^2+^ by destroying ATP-dependent Ca^2+^ channels in the smooth ER (SERCAs) and plasma membrane from clearing excessive cytosolic calcium. This results in the impairment of cytoprotective mechanisms such as autophagy and the unfolded protein response (UPR) that needs ATP (Biczo et al., 2018). Hence, this interaction enhances cell damage. Nevertheless, there still could be an abundant amount of ATP, which, if released, can play an essential role as DAMPs by activating purinergic signaling (Dixit et al., 2019b).

Once released, the massive accumulation of extracellular nucleotide could be regulated in the way of phosphorylation and dephosphorylation by triggering the activation of extracellular enzymes (ecto-enzymes). CD-39 mediates phosphohydrolysis of eATP (from ATP/ADP to AMP), which is subsequently converted to adenosine by CD73 (from AMP to adenosine) (Minor et al., 2019). CD73 has key role in maintaining homeostasis and regulates pathophysiological responses related to immune tolerance, inflammation, infection and cancer (Linden et al., 2019; Schneider et al., 2019; Vuerich et al., 2019). One study suggested that soluble CD73 is a biomarker of persistent organ failure and the severity of AP is better than C-reactive protein or creatinine (Maksimow et al., 2014). Nevertheless, there are still controversial attitudes toward this study (Sun and Messaris, 2014; Jiang et al., 2015). CD39 expression is regulated by several pro-inflammatory cytokines (such as IL-2, TGF-β), tissue damage, oxidative stress, tissue remodeling and hypoxia leading to the accumulation of eATP (Antonioli et al., 2013; Timperi and Barnaba, 2021). Künzli et al. found that CD39 deletion decreases fibrogenesis and alleviates inflammation in experimental pancreatitis (Kunzli et al., 2008). Another group of enzymes participated in the conversion eATP includes adenylate kinases (AKs) and the nucleoside diphosphate kinase (NDPK/NME) family, which *via* phosphohydrolysis to accelerates the transition from ATP to ADP (Zimmermann et al., 2012; Yegutkin, 2014).

eATP has been shown to be involved in guiding neutrophils to the site of injury, activating T cells, platelets, mast cells and monocytes, promoting inflammasome activation in macrophages, and releasing cytokine (Cekic and Linden, 2016). One study has shown that the release of eATP promotes systemic injury in severe acute pancreatitis (Dixit et al., 2019b). With the increasing concentration of eATP, purinergic signaling is triggered by nucleotides’ interaction in P2 receptors, such as transcell membrane cationic channels (P2XR) and G-protein coupled receptors (P2YR), thereby initiating autocrine/paracrine signaling, (Beavis et al., 2012; Zimmermann et al., 2012). P2X7 is a major stimulant of the intensity and duration of inflammation and immunity and is involved in the pathologies of cancer, nervous and cardiovascular diseases (Di Virgilio et al., 2017a; Adinolfi et al., 2018). By activating intracellular signal transduction pathways including NF-κB, nuclear factor of activated T cells (NFAT), PI3K-AKT-GSK-3b and hypoxia-inducible factor 1α (HIF1α), it promotes the release of various cytokines (such as IL-6 from human fibroblasts and TNF from human dendritic cells) and chemokines in different immune cells (Gong et al., 2020). Considered as one of the most potent activators of the NLRP3 inflammasome, the role of P2X7R for establishing an inflammatory response is widely studied in the activated innate myeloid cells (Martínez-García et al., 2019). Targeting P2X7/NLRP3 signaling pathway, a new study demonstrated that effectively alleviates ATP−induced pancreatic ductal cell injury to avoid ductal occlusion in AP by downregulating the protein levels of P2X7 (Zhang et al., 2019). Therefore, inhibition of the P2X7/NLRP3 signaling pathway could be a novel therapeutic target in AP.

### eCIRP and AP

Cold-inducible RNA-binding protein (CIRP) was discovered in the early twenty-first century during the research of mechanism of cold stress adaptation in mammals. Since then, the role of intracellular CIRP (iCIRP) as a stress-response protein has been extensively studied. In contrast, extracellular CIRP (eCIRP), an 172-amino acid RNA chaperone protein, was recently discovered to also act as DAMP. It plays an important role in pathobiology of inflammatory diseases (Aziz et al., 2019; Denning et al., 2019b). By drawing plasma at 24~48h after admission from patients with AP, the study found that in both mild and moderate severe/severe AP, the plasma levels of eCIRP were increased remarkably, and the results also suggest that eCIRP promotes systemic inflammatory responses to AP (Linders et al., 2020b).

The release of eCIRP can be divided into passive and active release. The eCIRP can be passively released by necrotic cells, but an *in vitro* study using macrophages suggests that passive release is not a major source of eCIRP during hypoxia or endotoxemia, and remains to be studied in AP (Aziz et al., 2019). Active release means that CIRP can migrate from the nucleus to the cytoplasmic emergency particles and then be released into the extracellular space through the lysosomal extracellular pathway during periods of cellular stress, such as hypothermia, hypoxia and hyperradiation (Denning et al., 2019b; Murao et al., 2021b).

**Figure 4:**
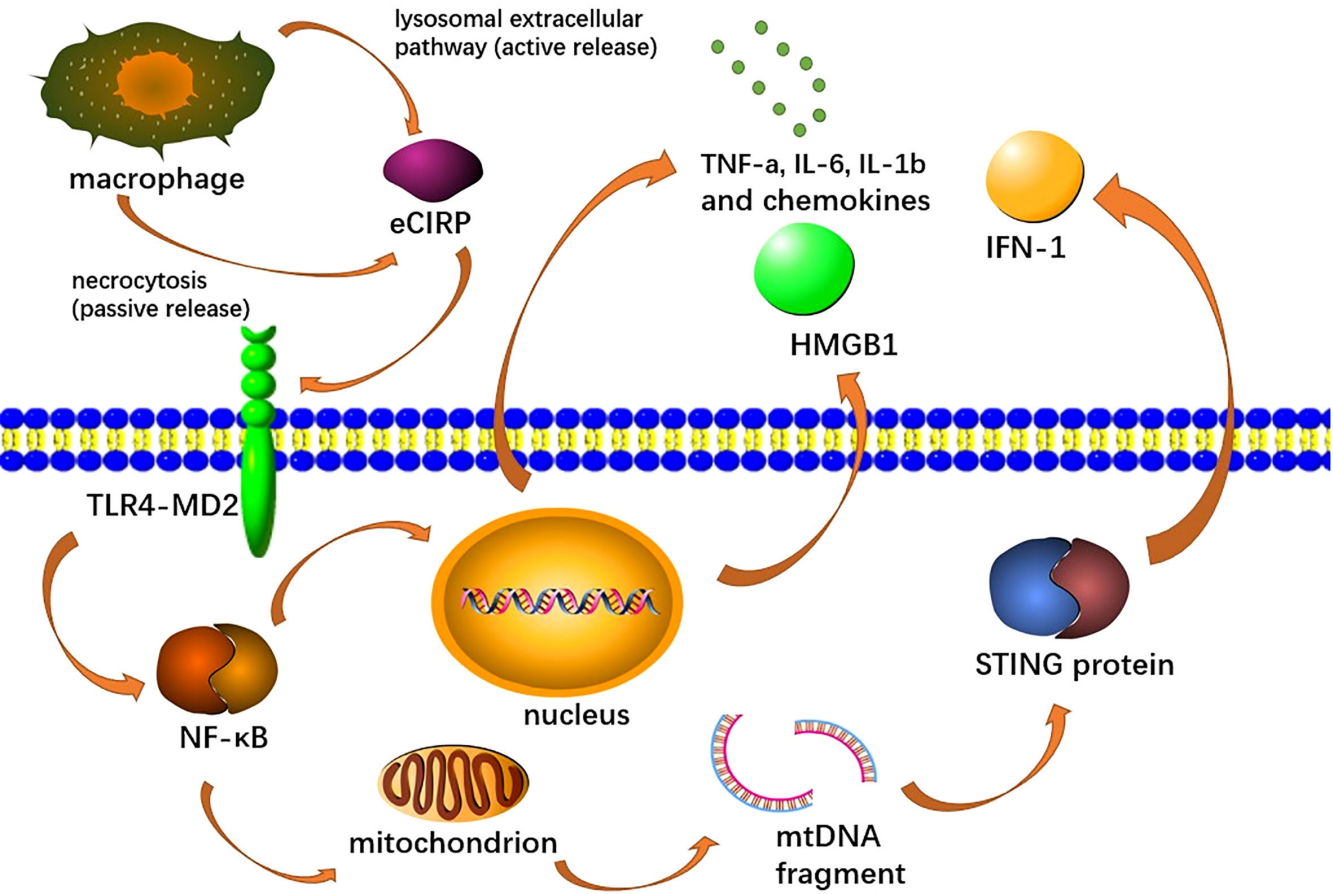
Extracellular CIRP (eCIRP) is released to the extracellular space *via* necrosis or lysozyme extracellular pathway, afterward binds to the TLR4-MD2 receptor complex on macrophages, activates the TLR4/MyD88/NF-κB pathway and induces macrophages to release pro-inflammatory cytokines (TNF-A, IL-6, Il-1b), chemokines (keratinocyte chemical attractor and MIP-2), and HMGB1. This pathway also promotes mitochondrial DNA damage and degradation, leading to STING activation, which leads to the production of type I IFN and pro-inflammatory cytokines.

As DAMPs, eCIRP induces endothelial cell activation, macrophage secretion of pro-inflammatory mediators and NETs formation (Qiang et al., 2013; Ode et al., 2019; Linders et al., 2020b). Among them, the function of inducing endothelial cell activation has been fully proved in the studies related to acute lung injury, but there is no relevant study in AP (Ode et al., 2019). It can activate the GSDMD (Gasdermin D) pathway of macrophages to promote MET formation, where GSDMDS is also a reforming protein involved in NET formation (Lee et al., 2021; Tan et al., 2022). Moreover, by recognizing TREM-1, an innate immune receptor expressed primarily in myeloid cells, it can activate macrophages (Denning et al., 2020; Murao et al., 2020; Lee et al., 2021; Murao et al., 2021b). eCIRP also binds to the TLR4-MD2 receptor complex, which activates the TLR4/MyD88/NF-κB pathway and induces macrophages to release pro-inflammatory cytokines (TNF-α, IL-6 and IL-1b) and chemokines (keratinocyte chemoattractant and MIP-2), as well as HMGB1 (**Figure 4**) (Linders et al., 2020b; Murao et al., 2021b). This pathway also promotes mitochondrial DNA damage and degradation, causing STING activation, which leads to the production of type I IFNs and pro-inflammatory cytokines, and has also been shown to activate NETs (Denning et al., 2019b; Gurien et al., 2020; Chen et al., 2021a; Chen et al., 2021b). In promoting the formation of NETs, eCIRP can induce the production of a different type of neutrophils, ICAM-1+ neutrophils, which can produce more NETs (Murao et al., 2020). Recent studies have also shown that eCIRP can also increase NET formation *in vivo* and *in vitro* by activating PAD4 (Denning et al., 2019b)

Inhibition of eCIRP has been shown to reduce NET formation, pro-inflammatory mediators and tissue damage in AP (Linders et al., 2020b). Qiang et al. developed a CIRP antagonist, C23, with a high affinity for the TLR4/MD2complex, where it reduces inflammation and tissue damage in sepsis, shock, and ischemia-reperfusion (Murao et al., 2021b). Moreover, C23-targeted eCIRP inhibits inflammation and tissue damage in AP (Linders et al., 2020b). Microrna130b-3p has been shown to inhibit ECIRP-mediated aseptic inflammation in the treatment of sepsis and acute lung injury, but its effect in AP has not been studied (Ode et al., 2019).

## NETs as extracellular fibrillar DNA networks

As the most abundant innate immune cells in human body, neutrophils play a key role in fighting bacterial infections and function mainly *via* three mechanisms, i.e. phagocytosis, degranulation and NET formation (Papayannopoulos, 2018; Wan et al., 2020). NETs are extracellular networks consisting of decondensed chromatin fibers studded with granular and cytoplasmic proteins and peptides (Brinkmann, 2018). NETs often contribute to pathogen clearance, but in excessive NETs can also lead to inflammation and tissue damage (Tan et al., 2021). DNA in NETs is mainly originated from the nucleus, but also mitochondria (Lood et al., 2016; Papayannopoulos, 2018). The proteins of NETs are mainly composed of histones as well as granular enzymes and peptides, among which are neutrophil elastase (NE), S100A8, lactoferrin, azurocidin, cathepsin G (CG), S100A9, myeloperoxidase (MPO), proteinase 3 (PR3), pentraxin 3, gelatinase, actin, lysozyme C, calprotectin, defensin, cathelicidin and catalase (Urban et al., 2009; Brinkmann, 2018). The release of NETs occurs primarily through a cellular death process termed NETosis (Papayannopoulos, 2018). There is a variety of inducing factors for NETosis such as infectious agents (viruses, bacteria, bacterial components, parasites, fungi), physiological stimuli (activated platelets, complement-derived peptides, antibodies, cytokines, microcrystals) and chemicals (hydrogen peroxide, tobacco smoke) (Brinkmann, 2018). There are two types of NETosis: suicidal NETosis, in which cells die, and vital NETosis, in which cells maintain certain viability and effector functions (Vorobjeva and Chernyak, 2020).

Suicidal NETosis, which lasts 2–4 h, begins with the activation of neutrophils upon the recognition of stimuli, mostly PMA, which induces the activation of the NADPH oxidase (NOX) complex and subsequent production of ROS through protein kinase C (PKC)/Raf/MERK/ERK (Masuda et al., 2016; Delgado-Rizo et al., 2017). The NADPH-dependent ROS production not only leads to chromatin decondensation by boosting the activation of calcium influx and peptidyl arginine deaminase 4 (PAD4) but also promotes the progressive nuclear membrane separation and loss (Delgado-Rizo et al., 2017; Denning et al., 2019b). The transport of elastase and myeloperoxidase from granules to nuclei also plays a key role in NET formation (Papayannopoulos et al., 2010). Cytoplasm and karyoplasm are mixed and finally released outside the cell through membrane pores and cellular lysis (Delgado-Rizo et al., 2017; Brinkmann, 2018).

Vital NETosis has been found out following specific microbe-associated molecular patterns (MAMP) recognized by host pattern recognition receptors, such as toll-like receptors, independent of ROS and the Raf/MERK/ERK pathway (Masuda et al., 2016; Delgado-Rizo et al., 2017). The induction of vital NETosis normally takes 30 minutes after PMN stimulation, as opposed to several hours for suicidal NETosis (Denning et al., 2019b; Zhou et al., 2020a). After the activation of vital NETosis, the nuclear envelopes dilates hugely and vesicles are formed (Brinkmann, 2018). Those DNA-containing vesicles concentrate near the plasma membrane and eventually fuse with it, releasing contents without cell lysis (de Buhr and von Köckritz-Blickwede, 2016; Brinkmann, 2018). Another type of vital NETosis independent of cell death while dependent of ROS has also been described, in which the mitochondrial DNA was released by neutrophils instead of the nuclear (Yousefi et al., 2009).

## NETs and AP

The important role of neutrophils in AP is well known, and Merza et al. demonstrated for the first time that neutrophils-derived NET is a core part of the pathophysiological process in severe AP (Merza et al., 2015). It has shown that the biomarker NET is not only present in AP but also associated with the severity of AP (Murthy et al., 2019). In septic AP, NETs fight infection by trapping and killing invading microorganisms (Pan et al., 2021). Neutrophils could cause partial and distal organ injury due to the release of NETs in severe AP (Korhonen et al., 2015).

NETs could participate in the pathogenesis of AP by induction of trypsin activation, damage of tissue and accelerating systemic inflammatory responses (Pan et al., 2021). In AP, premature activation of trypsinogen is considered to be a key factor in triggering the disease induction (Gui et al., 2020). Korhonen et al. proposed a convincing mechanism by which neutrophils activate intra-acinar trypsin (Korhonen et al., 2015). The study has shown that NETs are effective stimulators of macrophage-1 antigen (MAC-1) expression and ROS formation in neutrophils, and NETs can directly activate neutrophils (Merza et al., 2015). Inhibition of NETs resulted in a 93% reduction in matrix metallopeptidase-9 (MMP-9) circulating levels in taurocholic acid-exposed animals (Merza et al., 2015). Neutrophil-derived MMP-9 is important in AP as it is an effective trypsinogen activator and regulates trypsinogen activation and tissue damage (Awla et al., 2012). Signal transducer and activator of transcription-3 (STAT-3) is also a vital signal molecule of acinar cells (Madhi et al., 2019b). It has been found that NETs modulate STAT-3 activity and trypsin activation in acinar cells (Merza et al., 2015), and another study showed that the stimulation of NET could significantly enhance the liveness of STAT-3 (Madhi et al., 2019b). Therefore, NETs could activate trypsinogen through STAT-3, MMP-9, and other mechanisms, thus magnifying the extent of pancreatic damage in AP (Wan et al., 2020).

The initial injury in AP is sterile (Hoque et al., 2012), and Yang et al. firstly proposed an interesting new role for NETs in pancreatitis without microbiological infection (Yang et al., 2015). NETs have been shown to be key mediators of innate immune responses in aseptic inflammatory diseases (Murthy et al., 2019). Systemic inflammatory cascade activation is characteristic of severe AP and could result in systemic inflammatory response syndrome in pathological progression of disease (Hu et al., 2020). NETs not only cause damage to the pancreas, but also lead to severer damage to other involved organs, such as lung, blood vessel, kidney and heart damage (Hu et al., 2020). NETs mediate inflammation and thrombosis and play a role in the transitional period of hypercoagulability and severe inflammation in the incipient stage of acute pancreatitis (Wan et al., 2020). Formation of NET in inflammation has been shown to be highly pro-coagulant, due to exposure to cell free DNA (cfDNA), histones and neutrophil proteases, which leads to coagulation activation and inactivation of antithrombotic proteins, predisposed to formation of disseminated intravascular coagulation (DIC) (Liaw et al., 2016).

Neutrophils could cause acinar cell damage by activating trypsinogen and could directly cause acinar cell damage by secretion of ROS and MMP-9 (Saffarzadeh et al., 2012). NETs have cytotoxic effects (Saffarzadeh et al., 2012), and histones from NETs can cause acinar cytoplasmic leakage and cell death by disrupting the pancreatic acinar cell serosa (Hu et al., 2020). NETs on internal body surfaces are useful for monitoring potential hazards, but excessive formation of NETs may lead to epithelial cell damage (Leppkes et al., 2019). It has been reported that NET-derived histones could lead to damage and death of both epithelial and endothelial cells (Saffarzadeh et al., 2012).

Condensed layers of aggregated NETs can spatially screen and insulate the necrotic site, creating a temporary barrier to limit the spread of necrosis-related proinflammatory mediators (Bilyy et al., 2016). However, aggregated NETs could block secretion flow, driving focal pancreatitis and parenchymal remodeling (Leppkes et al., 2016). The production of intraductal aggregated NETs results in occlusion of the pancreatic duct and persistence of inflammation, leading to pancreatitis (Leppkes et al., 2016). NET is a double-edged sword, coordinating the innate immune response while it also carries the danger of precipitating autoimmunity and epithelial injury (Leppkes et al., 2019).

## Interaction between DAMPs and NETs

**Figure 5:**
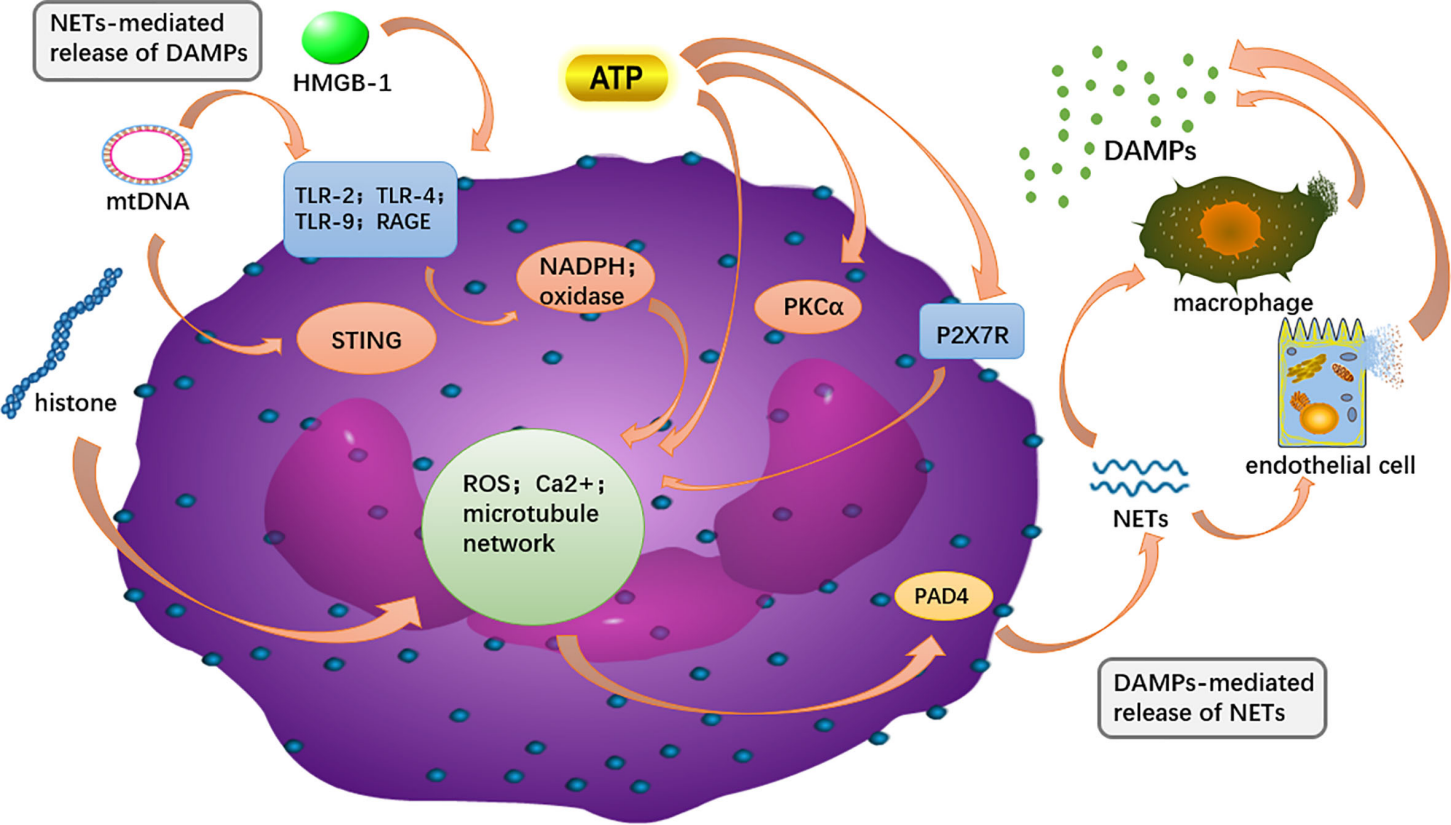
The intracellular DAMPs is released to the outside of the cell and can recognize PRR on surrounding neutrophils, and in turn, activate PAD4 to promote NET formation. NETs components, such as DNA and H3 histones, can further activate macrophages and endothelial cells, releasing more DAMPs out of the cell to amplify the inflammatory cascade.

Both NETs and DAMPs are closely associated with the development of AP, and there is growing support for a potential link between the two as well (**Figure 5**). NETs are covered by proteins such as histones, granulins and cytosolic proteins, and these major components are identified as DAMPs (Pires et al., 2016; Chapman et al., 2019). Some DAMPs acted as components of the NET enhance the inflammatory cascade by further stimulating immune cells as well as endothelial cells to release more DAMPs (Denning et al., 2019a). Correspondingly, various DAMPs have been shown to induce NETs.

HMGB-1 contributes to NET formation by attaching to receptors such as TLR2/4 and RAGE, which was reliant on NADPH oxidase (Ma et al., 2016; Wu et al., 2018a). NADPH oxidase, as an important source of ROS production, was engaged in HMGB-1-mediated activation of neutrophils (Xiang et al., 2011). Oxidative stress is thought to contribute to the formation of NETs, with HMGB-1 serving as a mediator for it (Wu et al., 2016). HMGB-1 induces NETosis, as reported in previous studies, including in hepatic I/R and LPS-injected animal models (Tadie et al., 2012; Huang et al., 2015). In liver I/R animal model, the authors found that the histone H3 of TLR4-KO neutrophils in response to HMGB1 and histones was reduced, with a more significant decrease observed in HMGB1-stimulated neutrophils. The result suggests that TLR4 is the predominant receptor for HMGB1 to form NETs (Huang et al., 2015).

In addition to its structural role as a component of NETs, mitochondrial DNA (mtDNA) activates neutrophils to release more NETs and participates in the inflammatory response (Yousefi et al., 2009; Balogh et al., 2013; Mcilroy et al., 2014). Neutrophils releasing mtDNA is ROS-dependent and independent of cell death, and mtDNA was shown to markedly induce extracellular DNA release (Yousefi et al., 2009; Liu et al., 2019). Research shows that mtDNA-induced NET formation is dependent on the both TLR9 and STING pathways (Liu et al., 2019). Liu et al. found that NET formation by mtDNA was suppressed in Sting-/- and TLR9-/-mice, indicating that the TLR9 and STING signal ways may exert its effects on the mtDNA-induced NET formation. In subsequent experiments, Sting-/-and TLR9-/- neutrophils exhibited decreased phosphorylation of extracellular signal-regulated kinases (ERK1/2) and p38 MAPK, and reduced levels of PAD4 and Ras-related C3 botulinum toxin substrate 2 (Rac2) in response to mtDNA. ERK1/2 and p38 MAPK are located downstream of the STING and TLR9 pathways. Therefore, when these pathways are inhibited, it results in a remarkable decrease in expression of PAD4 and Rac2 induced by mtDNA (Liu et al., 2019). Tumburu et al. suggested that a cytosolic pathway is responsible for mtDNA-induced NET formation and that serine/threonine TBK1 is involved in the process (Tumburu et al., 2021).

Citrullinated histones are key mediators in NET genesis and their co-localization with cytoplasmic components of neutrophils suggests the formation of NETs (Nakazawa et al., 2017a). Histone citrullination weakens the link between DNA and histones, ultimately leading to chromatin depolymerization and NET release. (Remijsen et al., 2011; Nakazawa et al., 2017b). Previous studies have demonstrated that histone H4 triggers NET formation *via* calcium and PAD4. Neutrophil membrane permeability and intracellular calcium content are continuously increased due to histone H4, it is a necessary condition for PAD4 activation (Shi et al., 2021). PAD4 promotes histone hypercitrullination, a primary precondition for NET formation, which in turn mediates chromatin depolymerization and facilitates the release of NETs (Li et al., 2010). Additionally, Hamam et al. showed that histone acetylation promoted NET formation by facilitating chromatin decondensation (Hamam et al., 2019).

The formation of NETs needs the genesis of ATP in the glycolytic system, reorganization of the cytoskeleton and ejective release of cytoplasmic granules along with mtDNA (Stojkov et al., 2017; Amini et al., 2018). NETosis mediated by ATP in blood polymorphonuclear leukocytes and bone marrow-derived PMN has been identified to occur by a mechanism involving Ca2+ influx by P2X7R, activation of PKC-α, elevated ROS genesis and subsequent increase of PAD4 (Kim et al., 2020). As is the case for HMGB1, DNA and histone, ATP is also an intracellular constituent released in the course of NETosis. During NETosis, numerous ectonucleotides (e.g. ATP, ADP, UDP, UTP and adenosine) are released, and it is speculated that there may be a vicious circle between NETosis and purinergic receptor-mediated autocrine responses to ectonucleotides (Sil et al., 2017; Kim et al., 2020).

## Application of DAMPs and NETs in AP therapy

Nowadays targeted therapeutic modalities against DAMPs are discussed mostly in four categories (**Table 2**):

**Table 2:** Intervention of DAMPs in AP therapy, with intervention type, targeted DAMPs and associated mechanism.

Intervention type	Substance	Targeted DAMPs	Mechanism	Reference
Decrease expression of extracellular DAMPs	LincRNA-EPS; Dexamethasone; Midazolam combined with sufentanil; pRNA-U6.1/Neo; Calycosin; Sodium Butyrate;microRNA-141; miR-340-5p; Danaparoid sodium	HMGB1	Suppress the HMGB1-NF-κB-dependent inflammation gene expression	(Hagiwara et al., 2009a; Hagiwara et al., 2009b; Zhang et al., 2010b; Luan et al., 2013; Zhang et al., 2015; Zhu et al., 2016; Zhao et al., 2018; Zhou et al., 2020b; Chen et al., 2021c; Zhu et al., 2021; Gao et al., 2022b)
Increase the expression of intracellular DAMPs	Hydrogen-rich gases	HSP	Increase the expression of HSP60 and resist the oxidation	(Li et al., 2021a)
	BRX-220	HSP	Increase the production of HSPs incluing HSP60 and HSP72	(Rakonczay et al., 2002)
	Bortezomib		Induce the pancreatic HSP72 and inhibit the proteosome	(Szabolcs et al., 2009)
	Insulin and insulin-mimetics	ATP	Upregulate glycolysis, prevent POA-induced ATP depletion, and inhibit Ca2+ overload	(Bruce et al., 2021)
	Cyclosporin A derivative (NIM811)		Serve as a cyclophilin D inhibitor to inhibit the opening of the mitochondrial	(Petersen et al., 2021)
Enhancing DAMPs elimination	HMGB1 neutralizing antibody	HMGB1	Bock high-mobility group box 1 and reduce the TLR4 and TLR9 expression	(Sawa et al., 2006; Chen et al., 2017)
	Heparin, Activated protein C, Thrombomodulin	Histones	Bind and inactivate histones	(Ammollo et al., 2011; Nakahara et al., 2013; Wildhagen et al., 2014)
	IRS954		Block TLR to reduce pancreatic edema and inflammatory	(Hoque et al., 2011)
Blocking DAMPs signaling	Protocatechuic acid; ALR;Abdominal paracentesis drainage;	HMGB1	Target HMGB1/TLR4/NF-κB signaling pathway	(Hagiwara et al., 2009a; Pan et al., 2018; Abdelmageed et al., 2021; Huang et al., 2021)
	Suramin	eATP	Block the P2 receptors and lead to reduced levels of plasma IL-6 and TNF-α	(Dixit et al., 2019a)

1. Decreased expression of extracellular DAMPs

2. Increased expression of intracellular DAMPs

3. Enhancing DAMPs elimination by receptor antagonists, antibody neutralization, competitive antagonism, etc.

4. Blocking DAMPs signaling

Although research has uncovered the involvement of NETs in the pathogenesis of sepsis, connective tissue diseases, cardiovascular diseases, autoimmune diseases and cancer, the investigation into the pathologies and promising therapeutic approaches of NETs in AP are still limited (Döring et al., 2017; Lee et al., 2017; Bonaventura et al., 2020; Masucci et al., 2020; Klopf et al., 2021; Cristinziano et al., 2022). Nowadays targeted therapeutic modalities against NETs mostly focusing on the intervention by the blockage of NETs formation (Madhi et al., 2019a; Madhi et al., 2019b; Madhi et al., 2019c; Murthy et al., 2019; Linders et al., 2020a; Wu et al., 2021). It could be worthy to explore more potential treatments such as facilitating the elimination of DAMPs **(** **Table 3** **)**.

**Table 3:** Intervention in NETs, and their mechanisms in AP therapy.

Substance	Mechanism	Reference
Chloroquine	Reduce serum cell-free DNA and citrullinated histone H3 in murine models of pancreatitis to regulate NET formation	(Murthy et al., 2019)
C3 gene-deficient	Disrupt complement cascades, reduce chemokine secretion, as well as decrease early infiltration of neutrophils into the pancreas and neutrophil extracellular traps formation through PAD4.	(Linders et al., 2020a)
GZD824	Abolish activation of c-Abl kinase to regulate NET formation, as well as decrease levels of citrullinated histone 3 in the pancreas and DNA-histone complexes in the plasma.	(Madhi et al., 2019a)
Cl‐amidine and GSK484	Reduce taurocholate‐induced increase of histone 3 citrullination in the pancreas and DNA‐histone complexes in the plasma to regulate NET formation	(Madhi et al., 2019c)
Platelet IP6K1 gene-deficient	Induce exogenous PolyP to regulate NET formation	(Madhi et al., 2019b)
PD1	Decrease early infiltration of neutrophils into the pancreas and neutrophil extracellular traps formation through PAD4.	(Wu et al., 2021)

Given the close relationship between DAMPs and NETs, the new approach targeting DAMPs and NETs could be promising and worth further investigation. However, until now, only one research from Wu focused on the close crosstalk between DAMPs and NETs in AP, revealing that HMGB1 could induce AP through activation of NETs and subsequent production of IL-1β, therefore a new therapeutic intervention could be attempted by targeting HMGB1 and NETs. Considering the rather limited research, further studies providing insights into the DAMPs and NETs in AP could be useful and are required.

## Conclusion

In this review, we gave an overview about the role of DAMPs and NETs in AP with concentrations on their mutual effects and therapeutic methods, in order to offer a better understanding of the pathophysiological mechanism and new insights for future investigational AP treatment options.

DAMPs are essential mediators in AP that may assume different roles depending on their location (intracellular vs. extracellular). While intracellular DAMPs may have protective value with regard to inducing autophagy that inhibiting the activation of the inflammasome, extracellular DAMPs may act as inflammatory factors through a variety of complex mechanisms. NETs could fight infection but also involved in the exacerbation of AP (Wan et al., 2020). The formation of NETs have been shown to be induced by variety of DAMPs. Both DAMPs and NETs have already been treatment targets for AP in many animal experiments.

However, new DAMPs are still being discovered and proposed definition and categories of DAMPs remain controversial. In addition, due to the complexity of the DAMPs pathways and the controversy of the NET formation, there are still many possible mechanisms and effects of DAMPs, NETs and their interactions that remain to be explored. With more precise mechanisms and newer DAMPs being proposed, further targeted and effective therapies can be established to minimize pancreatic injury and systemic inflammation, based on the information. They could also minimize damage to patient immunity. Although further discoveries are required, it is time to increase the development of the therapeutics/pharmaceutics and to put some fundamental findings into practice.

In conclusion, we performed a literature review on the role of DAMPs, NETs, and their interactions in AP. This could be useful for understanding of mechanism and prove noteworthy for people working in this area of research.

## Author contributions

XZ, SJ, JP, QL, SY, and WH conceptualized the study, searched and analyzed the literature, and wrote the draft of the manuscript. PA, ZB, VZ, and WH helped to finalize the manuscript. All authors listed have made a substantial, direct, and intellectual contribution to the work, and approved it for publication.

## Funding

This work was supported by Zhejiang Medical and Health Science and Technology Plan Project (Number: 2022KY886), Wenzhou Science and Technology Bureau (Number: Y2020010).

## Conflict of interest

The authors declare that the research was conducted in the absence of any commercial or financial relationships that could be construed as a potential conflict of interest.

## Publisher’s note

All claims expressed in this article are solely those of the authors and do not necessarily represent those of their affiliated organizations, or those of the publisher, the editors and the reviewers. Any product that may be evaluated in this article, or claim that may be made by its manufacturer, is not guaranteed or endorsed by the publisher.

## Abbreviations

DAMPs, Damage associated molecular patterns; NETs, neutrophil extracellular traps; AP, Acute Pancreatitis; ER, endoscoplasmatic retiuculum; NF- κB, nuclear factor-κb; RyR, ryanodine receptor; MPTP, mitochondrial permeability transition pore; SIRS, systemic inflammation reaction syndrome; LPS, bacterial lipopolysaccharide; PMA, phorbol 12-myristate 13-acetate; PKC, protein kinase C; PRR, pattern recognition receptors; NLR, nod like receptor; RIG-I, retinoic acid-inducible gene I; RAGE, receptors for advanced glycation end; HMGB1, high mobility group box-1; TLR, toll-like receptors; MALAT1, metastasis associated lung adenocarcinoma transcript-1; HSPs, heat shock proteins; HSF-1, heat shock factor protein 1; CD, cluster of differentiation; STING, (stimulator of the interferon gene); MAPKs, mitogen-activated protein kinases; MYD88, major reactive protein 88; cGAS, cyclic GMP–AMP; BMDCs, bone marrow-derived dendritic cells; SERCAs, channels in the smooth ER; UPR, unfolded protein response; AKs, adenylate kinases; NDPK/NME, nucleoside diphosphate kinase; NFAT, nuclear factor of activated T cells; HIF1α, hypoxia-inducible factor 1α; CIRP, cold-inducible RNA-binding protein; NE, neutrophil elastase; MPO, myeloperoxidase; PR3, leukocyte proteinase 3; NOX, NADPH oxidase; PAD4, peptidyl arginine deaminase 4; ROS, reactive oxygene species; MAC-1, macrophage-1 antigen; MMP-9, matrix metallopeptidase-9; cfDNA, cell free DNA; DIC, disseminated intravascular coagation; Rac2, Ras-related C3 botulinum toxin substrate 2; CitH3, citrullinated histone 3; PMNs, polymorphonuclear leukocytes; BM-PMNs, bone marrow-derived PMN.

## Glossary

**Table:**

DAMPs	Damage associated molecular patterns
NETs	neutrophil extracellular traps
AP	Acute Pancreatitis
ER	endoscoplasmatic retiuculum
NF- κb	nuclear factor-κb
RyR	ryanodine receptor
MPTP	mitochondrial permeability transition pore
SIRS	systemic inflammation reaction syndrome
LPS	bacterial lipopolysaccharide
PMA	phorbol 12-myristate 13-acetate
PKC	protein kinase C
PRR	pattern recognition receptors
NLR	nod like receptor
RIG-I	retinoic acid-inducible gene I
RAGE	receptors for advanced glycation end
HMGB1	high mobility group box-1
TLR	toll-like receptors
MALAT1	metastasis associated lung adenocarcinoma transcript-1
HSPs	heat shock proteins
HSF-1	heat shock factor protein 1
CD	cluster of differentiation
STING	(stimulator of the interferon gene)
MAPKs	mitogen-activated protein kinases
MYD88	major reactive protein 88
cGAS	cyclic GMP–AMP
BMDCs	bone marrow-derived dendritic cells
SERCAs	channels in the smooth ER
UPR	unfolded protein response
AKs	adenylate kinases
NDPK/NME	nucleoside diphosphate kinase
NFAT	nuclear factor of activated T cells
HIF1α	hypoxia-inducible factor 1α
CIRP	cold-inducible RNA-binding protein
NE	neutrophil elastase
MPO	myeloperoxidase
PR3	leukocyte proteinase 3
NOX	NADPH oxidase
PAD4	peptidyl arginine deaminase 4
ROS	reactive oxygene species
MAC-1	macrophage-1 antigen
MMP-9	matrix metallopeptidase-9
cfDNA	cell free DNA
DIC	disseminated intravascular coagation
Rac2	Ras-related C3 botulinum toxin substrate 2
CitH3	citrullinated histone 3
PMNs	polymorphonuclear leukocytes
BM-PMNs	bone marrow-derived PMN
